# Comprehensive analysis of glycerolipid dynamics during tobacco pollen germination and pollen tube growth

**DOI:** 10.3389/fpls.2022.1028311

**Published:** 2022-11-08

**Authors:** Natalia Serrano, Přemysl Pejchar, Hana Soukupová, Martin Hubálek, Martin Potocký

**Affiliations:** ^1^ Institute of Experimental Botany of the Czech Academy of Sciences, Prague, Czechia; ^2^ Institute of Organic Chemistry and Biochemistry of the Czech Academy of Sciences, Prague, Czechia

**Keywords:** lipidomics, phosphatidic acid, phospholipid, plasma membrane, pollen, pollen tube, tip growth, tobacco

## Abstract

Pollen germination and subsequent pollen tube elongation are essential for successful land plant reproduction. These processes are achieved through well-documented activation of membrane trafficking and cell metabolism. Despite this, our knowledge of the dynamics of cellular phospholipids remains scarce. Here we present the turnover of the glycerolipid composition during the establishment of cell polarity and elongation processes in tobacco pollen and show the lipid composition of pollen plasma membrane-enriched fraction for the first time. To achieve this, we have combined several techniques, such as lipidomics, plasma membrane isolation, and live-cell microscopy, and performed a study with different time points during the pollen germination and pollen tube growth. Our results showed that tobacco pollen tubes undergo substantial changes in their whole-cell lipid composition during the pollen germination and growth, finding differences in most of the glycerolipids analyzed. Notably, while lysophospholipid levels decrease during germination and growth, phosphatidic acid increases significantly at cell polarity establishment and continues with similar abundance in cell elongation. We corroborated these findings by measuring several phospholipase activities *in situ.* We also observed that lysophospholipids and phosphatidic acid are more abundant in the plasma membrane-enriched fraction than that in the whole cell. Our results support the important role for the phosphatidic acid in the establishment and maintenance of cellular polarity in tobacco pollen tubes and indicate that plasma membrane lysophospholipids may be involved in pollen germination.

## Introduction

In plant reproduction, the highly coordinated delivery of nonmotile sperm cells to the egg *via* a pollen tube represents a key innovation during the colonization of land by flowering plants because it allowed for sexual reproduction (Higashiyama and Takeuchi, 2015). Detailed knowledge of the mechanisms of sexual reproduction is critical for its obvious agronomic implications, but in addition, pollen germination and pollen tube growth are excellent model systems for understanding cell morphology. During pollen germination, processes determining the establishment of cellular polarity are overrepresented, and analogously, strictly apical elongation of pollen tubes involves many processes of cell polarity maintenance (Conze et al., 2017; Grobei et al., 2009; Hafidh and Honys, 2021). Typically, when pollen grain lands on the stigma (or is incubated in culturing medium), it rapidly hydrates to trigger a metabolic program for the germination stage. The hydration then switches on a signaling, metabolic and transcriptomic program that leads to the breakdown of energy stores, uptake of nutrients, reorganization of cytoskeleton and endomembrane organelles, cellular polarization, and buildup of vesicular trafficking machinery. After 30-60 min, this collectively leads to the emergence of a pollen tube from the germination pore, and a second developmental program focused on pollen tube growth gradually takes over (Wang et al., 2010).

There is tremendous membrane dynamics in germinating pollen and elongating pollen tubes, comprising both plasma membrane and various compartments of the endomembrane system (Zonia and Munnik, 2008). This coincides with the fact that pollen grains from all known species accumulate enormous amounts of glycerolipids, that are stored in endoplasmic reticulum-derived compartments termed lipid droplets (LD, Hernández et al., 2020). They provide ready-to-use material and the energy source for rapid membrane production and remodeling during germination and elongation of pollen tubes. It has been estimated that a pollen tube with a diameter of 10 μm would require around 2-3 pmol (corresponding to ~2 ng) of membrane lipids for each cm of length for the tonoplast and the plasma membrane alone (Ischebeck, 2016). All lipid classes, including sphingolipids, glycerophospholipids, galactolipids, triacylglycerols, sterols, and waxes, are present in pollen, where they are subject to tight regulation (Žárský et al., 2006; Ischebeck, 2016).

Glycerophospholipids are the most abundant type of phospholipids and constitute a crucial component of all eukaryotic membranes, including plants (Kerwin et al., 1994). Different glycerophospholipid families with distinct polar head groups bound to the sn-3 position of the glycerol backbone and diverse acyl-chain compositions are present in cell membranes. This headgroup and acyl chain composition of glycerophospholipids in various cell types, endomembrane compartments, and plasma membrane are quite distinct (Horn and Chapman, 2012; Nakamura et al., 2017). Based on the knowledge from mammalian and yeast studies, different lipid composition is also expected in the outer and inner leaflets of the plasma membrane (PM), with the glycerolipids dominating the in inner, cytoplasmic leaflet. However, direct evidence for PM asymmetry in plants is still missing (Cacas et al., 2016; Cassim et al., 2018). Importantly, this diversity and dynamic nature of membrane glycerolipids are not only due to *de novo* lipid biosynthesis in the ER and Golgi but also due to enzymatic activities of various phospholipases, lysophospholipid acyltransferases, and lipid kinases. This glycerolipid remodeling cycle thus contributes to the generation of membrane glycerophospholipid diversity and the production of lipid signaling molecules such as phosphatidic acid, phosphoinositides, lysophospholipids, diacylglycerol, and fatty acids (Colin and Jaillais, 2020; Heilmann and Heilmann, 2022).

Despite the apparent importance of membrane lipids in tip-growing pollen tubes, high-throughput lipidomic analyses in pollen are scarce compared to the wealth of data from sporophytic tissues. To some extent, pollen glycerolipids had been analyzed in rape seed (Piffanelli et al., 1997), lily (Nakamura et al., 2009), Arabidopsis (McDowell et al., 2013), wheat (Narayanan et al., 2018), tobacco (Krawczyk et al., 2022), and olive (Hernández et al., 2020). However, most of these studies studied non-germinated pollen grains or focused on pollen responses to heat stress. Here, we explored the glycerolipid composition of different stages of tobacco pollen germination and pollen tube elongation. Simultaneously, we analyzed the *in situ* activity dynamics of phospholipase A_2_, phospholipase D, and non-specific phospholipase C. To provide a glimpse into the compartment-specific lipid composition, we investigated the glycerolipid composition of the plasma membrane-enriched fraction. Having the advantage of tobacco (*Nicotiana tabacum* L.) pollen tubes as a model, which combines easy cultivation in quantities needed for various -omics experiments with genetic transformation and relatively high *in vitro* growth rates of 50-100 nm/s (Hepler et al., 2001; Chen et al., 2002), we also followed the dynamic localization of phosphatidic acid marker between various subcellular compartments.

## Materials and methods

### Pollen *in vitro* germination and lipid extraction

Tobacco (*Nicotiana tabacum *L. cv. Samsun) pollen was collected from the greenhouse-grown flowers and long-term stored at –20°C. For the lipid isolation at dedicated timepoints, pollen was resuspended in simple pollen growth medium (10% sucrose, 0.01% boric acid) at 2 mg/mL concentration, and 1 mL samples were immediately collected (timepoint 0’). After 30 minutes of imbibition, we collected the second set of 1 mL samples (timepoint 30’). Finally, after 3 hours, we collected the last set of 1mL samples (timepoint 3h). For each timepoint, five replicate cultures were cultivated.

Immediately after samples were collected, we performed the total lipid extraction using a slightly modified method of Bligh and Dyer (1959) using 4 mL of cooled (-20°C) methanol-chloroform 2:1 as described in Pejchar et al. (2010). The samples were centrifuged briefly and kept at room temperature for 30 minutes. Following, 1 M KCl was added to the mix, vortexed vigorously, and held at 4°C for 30 minutes. After 15 min centrifugation at 2000 x g, the organic phase was collected and transferred to a new 2 mL glass vial, dried with nitrogen, sealed with parafilm, and stored at -80°C.

Lipidome analysis was performed using an automated electrospray ionization-tandem mass spectrometry approach (Narayanan et al., 2016). The lipid extracts were introduced by continuous infusion into the ESI source on a triple quadrupole MS/MS (API 4000, Applied Biosystems, Foster City, CA). Samples were placed into the ESI using an autosampler compatible with the requirements of the ESI needle. Sequential precursor and neutral loss scans of the extracts generate a series of spectra with each spectrum revealing a set of lipid species containing a common head group. The background of each spectrum was subtracted, the data were smoothed, and peak areas integrated using an Applied Biosystems Analyst software (Xiao et al., 2010) in Kansas Lipidomics Research Center. The intensity values were calculated using a normalized intensity per mg pollen dry weight, and they were converted to the percentage of the total signal as it is the signal for each lipid species multiplied by 100 and divided by the total signal for the sample (Narayanan et al., 2018). All chemicals used were HPLC-grade.

### Plasma membrane isolation and characterization

For the isolation of the PM-enriched fraction, 0.15 g of dry pollen was germinated for 3 hours, and pollen tubes were collected by gentle filtration using a vacuum pump with a Miracloth filter. The sample was then split into two fractions, and the PM-enriched fraction was isolated with the Minute™ Plant Plasma Membrane Protein Isolation Kit (Invent Biotechnologies, Inc.). During the isolation, cytosolic and organelle fractions were also collected for monitoring purposes.

The isolated PM-enriched fraction (together with the cytosolic and organelle fraction) was separated on 10% SDS-PAGE gel and transferred to the PVDF membrane. After overnight incubation in 5% nonfat milk blocking solution (TBS with 0.05% Tween-20), the membrane was probed for 1 h with the following compartment marker antibodies (Agrisera) using the recommended dilutions: Anti-H^+^-ATPase (Plasma membrane maker, 90-95 kDa, AS07 260), Anti-UGPase (Cytoplasm marker, 52 kDa, AS14 2813), Anti-H3 (Nuclear marker, 17 kDa, AS10 710), Anti-BiP (Endoplasmic reticulum marker, 73.5/80 kDa, AS09 481), Anti-V-ATPase (Vacuolar marker, 26/31 kDa, AS07 213), and Anti-Arf1 (Golgi marker, 21 kDa, AS08 325). After washing, membranes were probed with the Anti-rabbit IgG secondary antibody (diluted 1:20000) conjugated with horseradish peroxidase (Promega). The detection was done with ECL™ Prime Western Blotting Detection Reagent (Cytiva) according to the manufacturer’s instructions.

### Sample preparation and mass spectrometry analysis

The samples for proteomic analysis were prepared in a 2% SDS buffer and heated up for 5 minutes at 95°C. The reduction was done using 100 mM Tris(2-carboxyethyl)phosphine (TCEP) for 30 minutes incubation at 37°C using the Microcon^®^ Filter Unit (UF). The filter placed on a collection tube was spun at 14000 g for 25 minutes. The collection tube was changed and 200 µL of buffer A (800 mM urea, 0.5 mM Deoxycholic acid (DCA), and 100 mM Ammonium bicarbonate (ABC)) were added. Tube was spun at 14000 g for 30 minutes. For the alkylation, we added 15 µL of 100 mM of Iodoacetamide (IAA) and 85 µL of buffer A to the filter and incubated for 30 minutes in the dark. Afterwards it was spun at 14000 g for 30 minutes. Following three washes with 3x100 µL of buffer A, the filter was centrifuged at 14000 g for 20 minutes. Additionally, the filter was washed three times with 100 µL buffer B (0.5 mM DCA, 50mM ABC) and centrifuged at 14000 g for 20 minutes. 100 µL of buffer B with trypsin (Pierce™ Trypsin Protease, MS-Grade), added to the UF device, was used for the digestion, which proceeded for 8 h at 37°C. Peptides were recovered by transferring the UF filter into a new collection tube and spinning at 14000 g for 15 min. To recover the peptides from the filter, 50 µL of 50 mM ABC was used twice and spun at 14000g for 15 minutes at RT. 200 µL of ethyl acetate, and 2.5 µL of TFA were added to the peptides and vortexed for 1 minute, followed by the addition of 1 mL of ethyl acetate and vortexed again. The tubes were spun at 16000 g for 10 minutes at RT. The organic phase was discarded, and these last two steps were repeated two more times. Peptides were evaporated in Speedvac (Erde et al., 2014).

The resulting peptides were separated on an UltiMate 3000 RSLCnano system (Thermo Fisher Scientific, Waltham, MA, USA) coupled to a Mass Spectrometer Orbitrap Fusion Lumos (Thermo Fisher Scientific) as described previously (Keilhauer et al., 2015; Langerova et al., 2020). The peptides were trapped and desalted with 2% acetonitrile in 0.1% formic acid at a flow rate of 30 µL/min on an Acclaim PepMap100 column (5 µm, 5 mm by 300-µm internal diameter (ID); Thermo Fisher Scientific). Eluted peptides were separated using an Acclaim PepMap100 analytical column (2 µm, 50-cm by 75-µm ID; Thermo Fisher Scientific). The 125 minutes elution gradient at a constant flow rate of 300 nL/min was set to 5% phase B (0.1% formic acid in 99.9% acetonitrile) and 95% phase A (0.1% formic acid) for the first 1 minute. Then, the content of acetonitrile increased gradually. The orbitrap mass range was set from m/z 350 to 2000 in the MS mode, and the instrument acquired fragmentation spectra for ions of m/z 100 to 2000. Proteome Discoverer 2.5 (Thermo Fisher Scientific) was used for peptide and protein identification using Sequest and Amanda as search engines against databases of tobacco downloaded from UNIPROT November 2021 and common contaminants. The mass spectrometry proteomics data have been deposited to the ProteomeXchange Consortium *via* the PRIDE (Perez-Riverol et al., 2022) partner repository with the dataset identifier PXD037046 and 10.6019/PXD037046.

### Statistical analysis

Various statistical analyses (including PCA analysis, One-way ANOVA with the *post hoc* tests, hierarchical cluster, and heatmap analyses) were done by the MetaboAnalyst web service (http://www.metaboanalyst.ca/). Significance was analyzed using ANOVA, FDR-adjusted *p* values lower than 0.05 were considered significant, and Tukey’s HSD was used as a *post hoc* test. We also used the web-based LipidSig tool for additional lipidomic data analysis and visualization of the plasma membrane and cell samples (Lin et al., 2021; http://chenglab.cmu.edu.tw/lipidsig/).

### *In situ* lipase analyses

Two microliters of the solution of fluorescently-labeled substrate Bodipy-phosphatidylcholine (2-decanoyl-1-(O-(11-(4,4-difluoro-5,7-dimethyl-4-bora-3a,4a-diaza-s-indacene-3-propionyl) amino) undecyl) sn-glycero-3-phosphocholine) (Bodipy-PC, D-3771, Invitrogen) in ethanol was added to the pollen culture (2 mg/mL) at indicated times (final concentration of Bodipy-PC was 0.66 µg/mL). After incubation on an orbital shaker at RT for 10 min, lipids were extracted as described above, the organic phase was evaporated to dryness by a vacuum evaporator, redissolved in ethanol and analyzed using thin-layer chromatography as described earlier (Pejchar et al., 2010). Briefly, samples were applied on the HP-TLC silica plates by automatic sampler, plates were developed in a mobile phase chloroform: methanol: water 65: 25: 4 (v/v/v), dried and scanned by FLA-7000 (Fujifilm) laser scanner. Individual spots were identified based on the comparison with fluorescently-labeled lipid standards and quantified by Multi Gauge (Fujifilm) software.

### Molecular cloning and stable tobacco transformation

Molecular cloning of the construct for genetically-encoded PA sensor (pUBQ::mCherry-NES-2xSpo20-PABD) was described previously (Kalachova et al., 2022). The final construct was transferred into *Agrobacterium tumefaciens* strain GV3101, which was used to transform tobacco (*N. tabacum *L. cv. “Petit Havana” SR1) plants by leaf-disk immersion method according to Horsch et al. (1985). Transformants were selected in kanamycin-containing medium.

### Confocal microscopy

For live-cell imaging, pollen and pollen tubes were observed with a spinning disk confocal microscope (Yokogawa CSU-X1 on Nikon Ti-E platform) equipped with a 60x Plan Apochromat objective (WI, NA = 1.2) and Andor Zyla sCMOS camera. Laser excitation (561 nm, laser box Agilent MLC400) together with 607/36-nm single-band filter (Semrock Brightline) were used for mCherry fluorescence collection.

## Results

**Figure 1:**
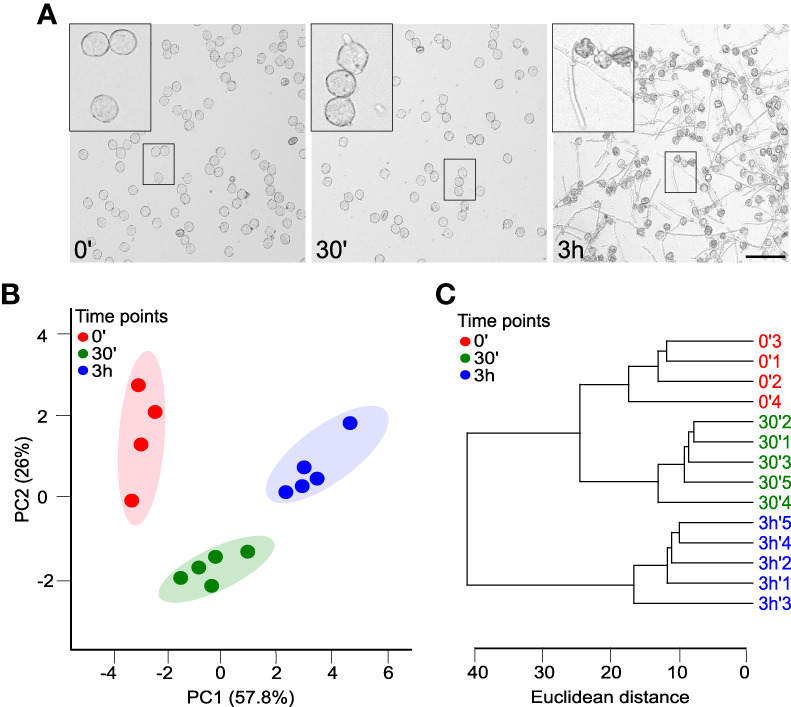
Tobacco pollen has a distinct whole-cell lipidome during the establishment and maintenance of cell polarity. **(A)** Time series of growing pollen tubes monitored at 0, 30 and 180 minutes after germination in liquid medium. Bar =100 µm. **(B)** Principal component analysis (PCA) of the glycerolipidome based on pollen tubes at 0, 30 and 180 minutes. The shaded areas indicate the 95% confidence regions. Samples are colored according to the time of collection 0’ (red), 30’ (green) and 3h (blue). **(C)** Dendrogram of hierarchical cluster analysis of individual glycerolipidome samples among pollen tubes at different times 0, 30 and 180 minutes. Both PCA and hierarchical clustering were done using the MetaboAnalyst package.

For successful delivery of the sperm cells, two main developmental switches must happen (i) pollen germination, i.e., the hydration of mature pollen and activation of processes leading to the emergence of a pollen tube; (ii) pollen tube growth, i.e., the successful maintenance of polarity-related processes ensuring the rapid tip growth, like the proper balance of anterograde and retrograde membrane trafficking, proper organization of actin cytoskeleton and the correct positioning of the male-germ unit. To monitor pollen lipidome changes in these crucial developmental steps, we sampled the *in vitro* tobacco pollen culture immediately after imbibition (timepoint 0’), 30 minutes after imbibition (timepoint 30’), and finally, after 3h of *in vitro* growth (timepoint 3h). These timepoints are well established as the most representative for pollen hydration, pollen germination, and pollen tube growth, respectively (Mascarenhas, 1975; Dorne et al., 1988). The microscopic monitoring confirmed that at 0’, >90% of pollen grains were correctly hydrated, germination was occurring *en masse* at 30’, and almost all pollen grains developed nicely growing pollen tubes at 3h (**Figure 1A**).

### Glycerolipidome undergoes substantial changes during tobacco pollen germination and pollen tube elongation

Recommended plant lipid isolation procedures typically involve solid samples (e.g., leaves), and great caution was made to prevent artificially high levels of phosphatidic acid (PA) caused by an uncontrolled release of phospholipase D (PLD). To inactivate PLD and other lipolytic enzymes, sample incubation in hot isopropanol with 0.01% BHT, followed by a mixture of chloroform-methanol-water, is recommended as the first isolation step (Shiva et al., 2018). We initially attempted to isolate the lipids from liquid pollen tube culture using this protocol, which required the rapid removal of the medium prior to hot isopropanol addition. Unexpectedly, our pilot results showed atypically high levels of PA (~40%) at the expense of phosphatidylcholine (PC) and phosphatidylethanolamine (PE) levels, likely caused by the cell damage (**Supplementary Figure 1**). Therefore, we reverted to the classical lipid extraction technique that uses a 2:1 ratio of methanol: chloroform as a first step (Bligh and Dyer, 1959) and is compatible with the liquid cell culture samples. This isolation approach, followed by the electrospray ionization‐tandem mass spectrometry, quantified 115 lipid analytes from 11 glycerolipid classes (**Supplementary Table 1**). These included two galactolipids class lipids – 8 species of monogalactosyldiacylglycerol (MGDG) and 9 digalactosyldiacylglycerols (DGDG). Among glycerophospholipids, we identified 4 species of lysophosphatidylglycerol (LPG), 6 lysophosphatidylcholines (LPC), 4 lysophosphatidylethanolamines (LPE), 18 phosphatidylcholines (PC), 21 phosphatidylethanolamines (PE), 10 phosphatidylglycerols (PG), 14 phosphatidylinositols (PI), 12 phosphatidylserines (PS) and 9 phosphatidic acids (PA).

To further explore the difference between the time points and get a comprehensive view of the lipidomic data, we performed a multivariate analysis on the dataset employing the MetaboAnalyst 5.0 pipeline (http://www.metaboanalyst.ca; Pang et al., 2022), which is also geared towards the processing of lipidomics data (Narayanan et al., 2018). Principal component analysis (PCA) of the pruned and scaled data (**Supplementary Table 2**) shows that replicates from each timepoint are grouped together and that the datasets from individual timepoints are well-separated, with the first two principal components explaining 84% of data variation (**Figure 1B**). In parallel with the PCA analysis, the hierarchical clustering of the data corroborated the similarity of the replicates for each timepoint and suggested that general glycerolipidome profiles for hydrated and germinated pollen are more closely related compared to pollen tubes (**Figure 1C**).

**Figure 2:**
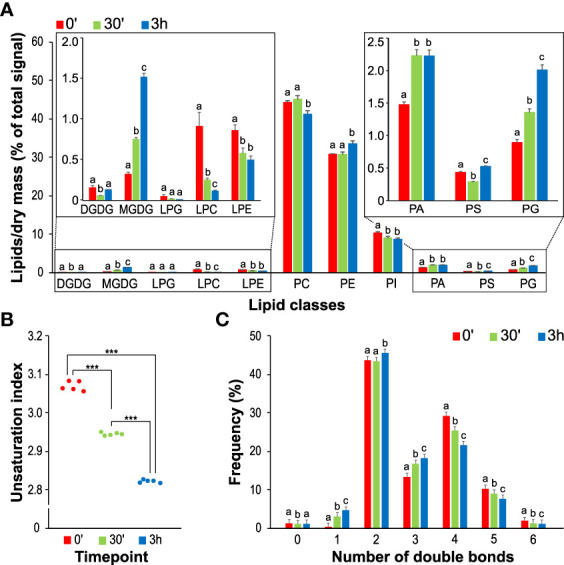
Changes in glycerolipid classes during the process of pollen germination and pollen tube elongation. **(A)** The composition of the main lipid classes (DGDG, MGDG, PG, PC, LPG, LPC, LPE, PE, PI, PS and PA) at different time points during the germination process of pollen tubes. The inset shows the composition of selected lipid classes in more detail. Data are presented as mean ± sd, n=4 (0’) and n=5 (30’ and 3h). Different letters indicate significant differences between samples at *p* < 0.05. **(B)** Changes of total unsaturation index during the different timepoints. **(C)** Double bonds distribution in the different time points. Asterisks (***) represent the significance between samples at *p* < 0.001.

The overall analysis of the pollen glycerolipid classes showed that the non-charged zwitterionic phospholipids PC and PE constitute most of the pollen glycerolipids with ~50% and ~35%, respectively (**Figure 2A**). Among negatively charged phospholipids, PI species in pollen comprise ~10%, while the total PA levels reach 1.5%, the total amount of PG is 0.9%, and PS is present at <0.5%. Lysophospholipids LPC and LPE represent ~0.8% of total glycerolipids each, and LPG was found only in trace amounts. Finally, galactolipids MGDG and DGDG are the minor components of the pollen glycerolipidome, constituting 0.3% and 0.15%, respectively. These relative phospholipid proportions (i.e., PC~PE>PI>PA~PG>PS) corresponded somewhat to glycerolipid compositions in non-photosynthesizing sporophytic tissues, such as Arabidopsis or tomato roots (Devaiah et al., 2006; Pfaff et al., 2020). Similarly, the presence of low galactolipid levels is consistent with the previous reports showing that although pollen and pollen tubes do not develop photosynthesizing chloroplasts, MGDG and DGDG were also detected in Arabidopsis, lily, and wheat pollen (Nakamura et al., 2009; McDowell et al., 2013; Narayanan et al., 2018).

Notably, all analyzed pollen glycerolipid classes underwent significant and distinct changes during the pollen hydration-to-germination and/or pollen tube germination-to-elongation switch (**Figure 2A**). Surprisingly, this was also true for bulk phospholipids like PC and PE, which displayed the opposite trend during pollen tube growth. Also, both detected galactolipids (MGDG and DGDG) showed significant but unrelated changes, where total MGDG levels continuously increased with time while the amount of DGDG transiently decreased during germination. Inverse dynamics was also observed for anionic lipids PA and PI, where relative PA levels go up during the germination while PI decreases. The dynamics of PG strongly resembles that of MGDG, while PS shows a minor decrease during the germination phase. Finally, all lysophospholipid levels seem to decrease during the germination and pollen tube phase, with LPC showing the most notable changes.

Next, we checked the general unsaturation index and the changes in saturated, monosaturated on polyunsaturated lipids in our datasets. We observed a significant decrease in unsaturation during both pollen germination and pollen tube elongation (**Figure 2B**), which is most prominent in glycerolipids with polyunsaturated fatty acids (PUFAs), i.e., those with the total number of double bonds >4 (**Figure 2C**). This is accompanied by an increase in monounsaturated lipids. Those general trends are driven by bulk phospholipids PC, PE, and PI, but they can also be seen in the extreme form in glycolipids, where DGDG and MGDG in hydrated pollen contain almost exclusively two PUFAs (i.e., containing 6 total double bonds) (**Supplementary Figure 2**). It is worth mentioning that these findings correspond well with the analysis of Arabidopsis cell culture when fast-growing cells also tended to accumulate monosaturated lipids and reduce the content of PUFAs (Meï et al., 2015).

### Alteration of lipid species composition during pollen germination and pollen tube growth

**Figure 3:**
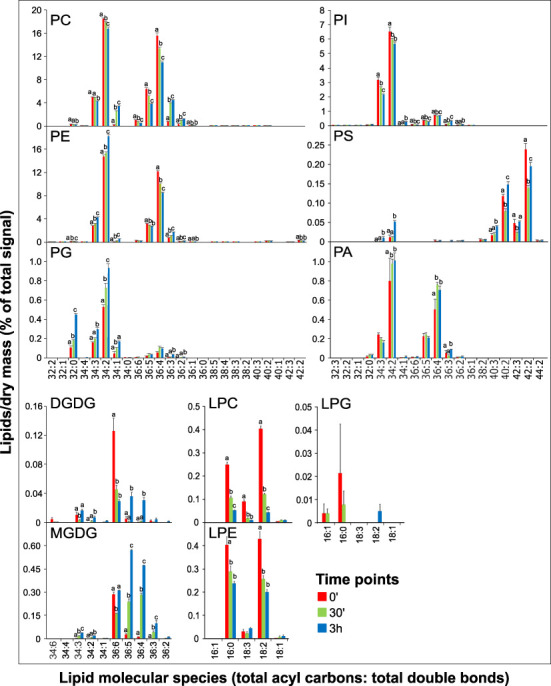
Changes in lipid molecular species during tobacco pollen germination and pollen tube elongation. Data are presented as mean ± s.e.m., n=4 (0’) and n=5 (30’ and 3h). Different letters indicate significant differences between samples at *p* < 0.05.

To get more specific insight into the glycerolipid changes during the switch from hydrated to germinated pollen and elongated pollen tubes, we analyzed the changes in individual lipid species. The composition of phospholipids in all three stages was dominated by two species, 34:2 and 36:4 (**Figure 3**). Although our analysis did not allow for direct identification of individual fatty acids (FAs), based on the lysophospholipid profiles, we can estimate that 34:2 phospholipids predominantly contain palmitic (16:0) and linoleic (18:2) acids. At the same time, 36:4 lipids are composed primarily of two 18:2 FAs and, to a much lesser extent, of oleic (18:1) and linolenic (18:3) acids (**Figure 3**). Analogously, we can estimate the FA composition of other significantly occurring phospholipids; 34:3 contains mainly 16:0/18:3, 36:3 contains mainly18:1/18:2 or 18:0/18:3, and 36:5 consists of 18:2/18:3.

Regarding the dynamics of specific phospholipid species, complex patterns can be seen for most phospholipids. For example, the most abundant species of PC, 34:2 and 36:4, both gradually decrease in time, whereas PE 34:2 and PE 36:4 display opposite trends. The same pattern is evident for 34:3, where PE increases during pollen germination and tube elongation while levels of PC 34:3 go down. Thus, PE 34:2 and 34:3 are mainly responsible for the increase in total PE levels (**Figures 2A**, **3**). Notably, both PC 34:1 and PE 34:1 are massively boosted during pollen germination (over 10-fold and 5-fold increase, respectively), and similar dynamics was recorded for PC 36:3 (5-fold accumulation). On the other hand, most PI species decreased, including the most abundant PI 34:3 and PI 34:2, and a slight increase was only observed for monosaturated PI 34:1 and for PI 36:3. PG species then all follow the same linearly increasing trend. Among minor signaling phospholipids, both dominant PA species, PA 34:2 and PA 36:4, show a modest but significant increase in the pollen germination phase, while their relative levels do not further increase during pollen tube growth. Conversely, PS molecular species show distinct trends in both acyl-chain composition and relative changes during pollen germination and growth, which are unapparelled with other tested glycerolipids (**Figure 3**). First, predominant PS species PS 40:2, PS 40:3, PS 42:2, and PS 42:3, containing very-long-chain fatty acids (VLCFAs), together constitute >90% of total pollen PS, which contrasts with reported values for Arabidopsis (McDowell et al., 2013), wheat (Narayanan et al., 2018) and tobacco (Krawczyk et al., 2022) pollen. Second, PS species show unique dual dynamics, where particularly shorter species (PS 34:3 and 34:2) are significantly upregulated in the pollen tube phase. In contrast, most VLCFA-containing PS species exhibit 30-50% drop during the pollen hydration-to-germination phase and increase again during pollen tube elongation.

Although they are present at low absolute values, lysophospholipids also displayed profound changes in the two pollen developmental switches. Both major LPC and LPE species (16:0 and 18:2) decreased rapidly during pollen germination, partly reflecting the changes in PC and PE with 34:2 or 36:4 composition (**Figure 3**). Interestingly, while LPC 18:3 levels also sharply fall during the germination phase (5-fold decrease), LPE 18:3 remains constant in all three pollen phases, suggesting a distinct function of these polyunsaturated lysophospholipids species. Finally, despite the different behavior of the two detected galactolipids, MGDG and DGDG (**Figure 2A**), analysis of their molecular species during pollen germination and pollen tube growth suggested certain common trends. In hydrated pollen, single polyunsaturated species (MGDG 36:6 and DGDG 36:6) make up the absolute majority of galactolipids (85% and 90%, respectively), and they are progressively desaturated to 36:5, 36:4, and 36:3 (**Figure 3**).

### Correlated lipid patterns through pollen germination and pollen tube elongation *in vitro*

To identify lipid clusters that follow specific patterns during the switch from hydrated pollen to the germinating one and/or growing pollen tube, all detected glycerolipids species were hierarchically clustered using the MetaboAnalyst service. We focused on eight different patterns representing different dynamic behaviors in the three time points (e.g., continuous increase or continuous decrease, etc., **Supplementary Figure 3**). In total, 70 lipids (i.e., 61%) showed some dynamic behavior, with the majority (43 or 37%) exhibiting upward trends. While this can be partly explained by an overall increase in overall metabolic activity, the lipid class population of these clusters differs significantly. PC, PE, and PG species make up most of the continuous increase cluster, while the abundant cluster (no change during germination followed by an increase in the pollen tube phase, 19 lipids) is populated predominantly by minor lipids like PS and DGDG (**Supplementary Figure 3**). PA, PC, and PE are then present in the cluster with an increase during germination and stable levels in the pollen tube phase. The three downward trends were followed by 23 lipids (20%); as suggested by **Figures 2A**, **3**, lysophospholipid and PI species are strongly represented here. Finally, only 5 lipids (4.3%) displayed transient increase or decrease in the germination phase, with the PUFA-containing PS species being the most interesting lipids (**Supplementary Figure 3**).

### Lysophospholipids and phosphatidic acid are enriched in pollen tube plasma membrane

**Figure 4:**
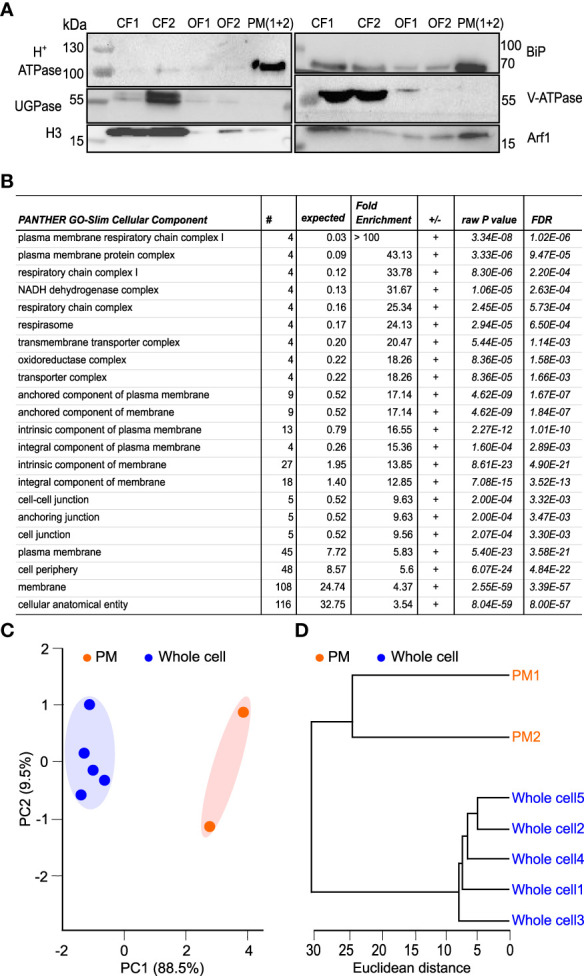
Proteomic and lipidomic characterization of plasma membrane-enriched fraction from tobacco pollen tubes. **(A)** Plasma membrane isolation. PM was isolated from germinated pollen tubes after 3h of germination in liquid medium. Western blots were performed to prove the enrichment of PM in the samples, using the different fractions obtained during the isolation protocol. CF represents cytosolic fraction (1 and 2), OF represents organelle membrane fraction (1 and 2) and PM represents the plasma membrane (combination of 1 and 2 fractions as suggested in the protocol). Cellular compartment markers used -H+-ATPase (Plasma membrane), UGPase (Cytoplasm), H3 (Nucleus), BiP (Endoplasmic reticulum), V-ATPase (Tonoplast-Vacuole) and Arf1 (Golgi apparatus). **(B)** Gene ontology (GO) analysis of the plasma membrane-enriched fraction showing the GO-slim cellular component significantly overexpressed in our proteomics results. **(C)** Principal component analysis (PCA) scores plot of the glycerolipidome extracted from whole-cell and PM enriched fraction. The explained variances are shown corresponding axes and the shaded areas indicate the 95% confidence regions. Samples are colored according to sample origin: whole cell (blue) and PM (orange). **(D)** Dendrogram of hierarchical cluster analysis of individual glycerolipidome from whole cell and PM. Both PCA and hierarchical clustering were done using the LipidSig package.

After 3 hours, pollen tubes have significantly increased their surface, accompanied by a concomitant increase of cellular membranes, including the plasma membrane (Mascarenhas, 1975). We, therefore, set out to investigate the distinct lipidomic features of pollen tube plasma membrane (PM) isolated from the 3h-old cells and compared them to the whole cell samples. To achieve this, we prepared PM-enriched fractions, employing a commercially available kit that allows the isolation of PM even from relatively low starting points. We tested the quality of the obtained fractions with Western blot, utilizing the battery of compartmental markers. Our data demonstrate a significant enrichment of the plasma membrane in the isolated fractions, although the presence of some endomembranes, in particular, ER and Golgi, was also detected (**Figure 4A**). To further corroborate this, we analyzed our fractions using the proteomic approach. After filtering out cytoplasmic contaminants, we found 269 peripheral and integral membrane proteins reliably identified in both PM preparations (**Supplementary Table 4**). Gene ontology analysis of the identified membrane proteins has corroborated our Western-blot data and showed that plasma membrane-related GO terms were significantly overrepresented, demonstrating plasma membrane enrichment in the purified fractions (**Figure 4B**). We then isolated glycerolipids from the PM-enriched fractions (further shortened to PM for brevity) and performed a comparative analysis with the whole cell glycerolipidome, replicating the strategy described above.

**Figure 5:**
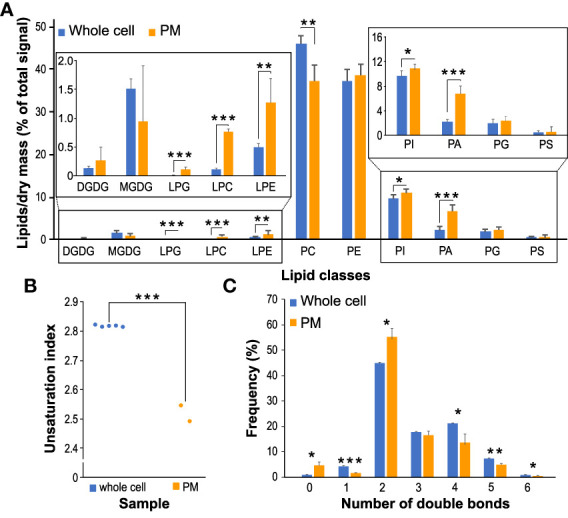
Changes in glycerolipid classes between PM-enriched fractions and corresponding whole cell samples. **(A)** The composition of the main lipid classes (DGDG, MGDG, PG, PC, LPG, LPC, LPE, PE, PI, PS and PA) for whole cell and PM samples. Data are presented as mean ± sd, n=5 (whole cell) and n=2 (PM). Asterisks (*), (**), (***) represent the significance between samples at *p *< 0.05, *p* < 0.01 and *p* < 0.001, respectively. **(B)** Total unsaturation index between PM and whole cell datasets. **(C)** Double bonds distribution between PM and whole cell datasets.

PCA analysis demonstrated that replicates from PM fractions are separated from the whole cell replicates, with the first PC (principal component) explaining almost 89% of total variation (**Figure 4C**). Supplementary to the PCA analysis, results from the hierarchical clustering (**Figure 4D**) confirmed the resemblance between each set’s replicates and the distance between the two datasets. The comparison of the lipid classes between the two datasets revealed no significant changes in galactolipids in PM-enriched fractions compared with the whole cell samples. Similarly, relative PS, PG, and PE levels remain the same in PM as in the whole cell membranes. While PI levels were only slightly increased in the PM, PA levels in the PM samples reached 7%, more than a three-fold increase compared to the whole cell membranes. Somewhat unexpectedly, all detected lysophospholipids were markedly increased in the PM dataset, with LPC showing seven-fold enrichment (**Figure 5A**). The unsaturation index of PM is significantly lower than the whole cell sample, in agreement with most eukaryotic systems (**Figure 5B**). Notably, an increase in saturated and a decrease in monounsaturated lipids is apparent in the PM dataset (**Figure 5C**). This is consistent with the lower propensity of general PM for extreme curvatures which is typical for endomembranes and is characterized by increasing monounsaturated lipids at the expense of saturated ones (Antonny et al., 2015).

**Figure 6:**
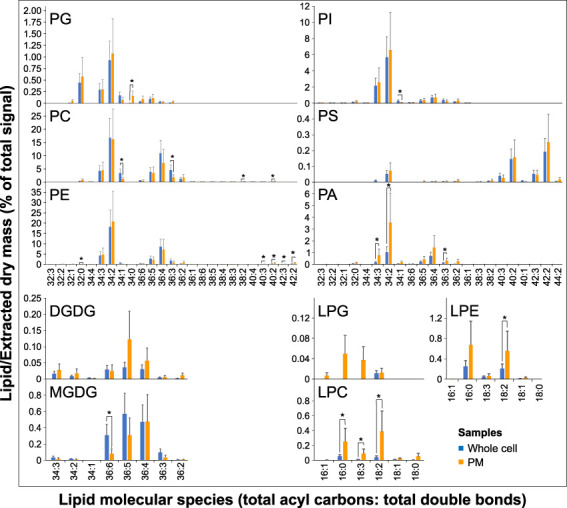
Changes in lipid molecular species between PM and the whole cell. The levels are normalized. Data are presented as mean ± s.e.m., n=5 (whole cell) and n=2 (PM). Asterisk represent the significance between samples at *p *< 0.05.

A comparison of molecular lipid species between PM and whole cell samples did not reveal striking differences in the PM’s galacto- and phospholipid composition, and majority of the most abundant species in each class show the same relative abundance (**Figure 6**). The increase of saturated lipids in the PM can be attributed to PG 34:0, which was not detected in the whole cell samples; similarly, the decrease of monosaturated phospholipids in the PM is due to PC 34:1 and PI 34:1. The distribution of phosphatidic acid species suggests that PA 34:2, PA 34:3, and PA 36:3 are boosted in the PM, with the relative abundance shifted from longer to shorter species (**Figures 3**, **6**). Notable changes can be seen for lysophospholipids, where polyunsaturated LPCs (18:2 and 18:3) significantly increased in the PM (**Figure 6**).

### *In situ* phospholipase A_2_ and D activities alter during pollen germination

**Figure 7:**
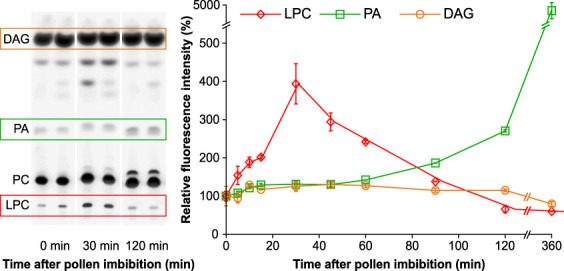
Lipid dynamics during pollen tube germination and pollen tube growth monitored by *in situ* analysis of PLD, PLA_2_ and NPC activity. Left, profiles of Bodipy-labeled lipid products of pollen culture at 0, 30 and 120 minutes after imbibition. Lipids were extracted after 10 min of incubation and separated by high-performance thin layer chromatography (HP-TLC). LPC, PA and DAG are boxed. Representative cut-outs from 4 independent experiments performed in duplicates are shown. Right, quantification of individual Bodipy-labeled lipid products. Data are presented as mean ± s.e.m., n=8.

To independently study the dynamics of phospholipid turnover in the pollen germination and pollen tube elongation phases, we used a fluorescent derivative of PC (Bodipy-PC) as a phospholipase substrate, which we established in the pollen tube system previously (Pejchar et al., 2010). This experimental setup, based on short-term incubation of pollen culture with Bodipy-PC (at different times after the imbibition) and followed by lipid extraction and thin layer chromatography separation, allowed us to directly monitor the activities of phospholipases A_2_ (PLA_2_), phospholipases D (PLD) and non-specific phospholipases C (NPC), which generate LPC, PA, and diacylglycerol (DAG), respectively. **Figure 7** shows that Bodipy-LPC production (reflecting PLA_2_ activity) is indeed linked with pollen germination, peaking 30 minutes after imbibition, while PLD activity increases steadily in growing pollen tubes. Interestingly, the DAG levels produced by NPC family members remain constant during all germination and tip growth phases. Taken together, the *in situ* analysis of LPC and PA production independently corroborates the role of lysophospholipids in the first 30 minutes of pollen germination.

### Spatiotemporal analysis of phosphatidic acid during pollen tube germination and elongation

**Figure 8:**
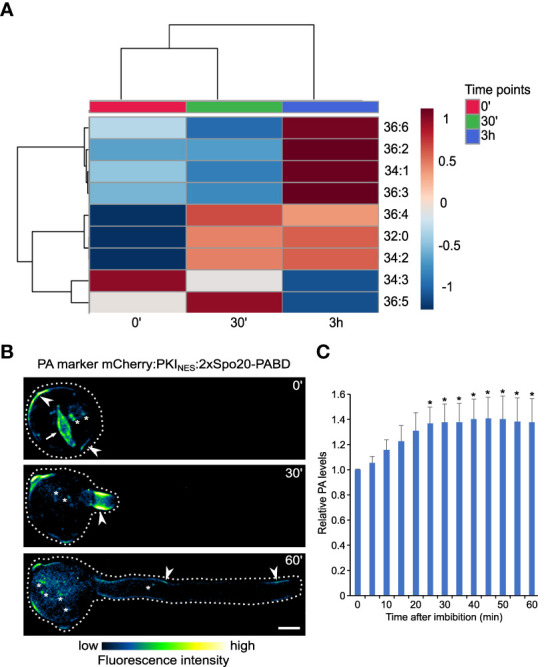
Distinct PA pools are mobilized during pollen germination and pollen tube growth. **(A)** Heatmap showing the correlation among phosphatidic acid (PA) species of germination and elongation of pollen tube samples. Data of the % of total signal of each species (identical to the data presented on Fig. 3) were normalized and analyzed using Spearman rank correlation analysis within MetaboAnalyst. Each colored cell on the map indicates the correlation coefficient, with the scale code shown on the top right corner. Blue and red colors on the heatmap indicate negative and positive correlations, respectively for increasing and decreasing species abundance relative to the mean of the 3 sample groups in the row for the 0’, 30’ and 3h sets, respectively; the color tint indicates fold-changes up to one and down to -1. The white color stands for no change, and gray for not detected. **(B)** During pollen germination and tube elongation, genetically-encoded PA markers relocalizes between different regions of pollen plasma membrane (arrowhead), nuclear membranes (arrow) and endomembranes (asterisk). Spinning disk confocal imaging (average Z-projections) of germinating tobacco pollen stably expressing PA marker mCherry-NES-2xSpo20-PABD under the control of UBQ10 promoter. Pollen was imaged at indicated times after imbibition and a cell exhibiting typical localization pattern (n=12) is shown. **(C)** Quantitative microscopic analysis of PA levels across all cellular pools during pollen germination and growth. Total fluorescence levels of PA marker were measured in ROIs encompassing the whole cell (dashed line) at indicated times after pollen imbibition. Relative fluorescence (normalized to time 0) is shown as mean ± SE (n=12 from three independent experiments), asterisk indicates time points different from time 0 (one-way Anova followed by Dunnet’s test, *p*<0.05).

Our results suggest that PA may be vital in establishing cellular polarity, exemplified by the process of pollen germination. Moreover, PA is quantitatively the most enriched glycerolipid in the PM, and almost 40% of the PA species are significantly higher in the PM fraction. To see which PA species are putatively linked with either pollen germination or pollen tube elongation, we reanalyzed data from **Figure 3** and performed a clustering of all PA species in the three timepoint samples. This approach yielded three clusters and suggested that in addition to major PA species (PA 34:2, PA 36:4, and also PA 32:0) that increase mainly during germination, there is a cluster of minor PA species that are primarily produced during pollen tube growth (PA 36:2, PA 36:3, PA 36:6, and PA 34:1; **Figure 8A**).

Next, we tested the changes in global PA levels in the pollen germination and pollen tube elongation phases, using tobacco pollen stably expressing genetically-encoded PA sensor (NES-2xSpo20-PABD) fused to mCherry fluorescent protein. This allowed us to assess the changes in PA levels semi-quantitatively in time and analyze spatiotemporal dynamics of different subcellular PA pools. We noticed that in the hydrated pollen, PA decorated mainly the pollen endomembrane surrounding the generative cell and compartments associated with the vegetative nucleus (**Figure 8B**). Upon germination, the endomembrane signal was rapidly lost and/or relocalized to the subapical plasma membrane domain of the emerging pollen tube, where it remained relatively stable during the subsequent elongation. Interestingly, the quantification of the total fluorescence signal of the PA marker over time somewhat resembled the lipidomic data, suggesting that a major pool of plasma membrane PA is formed during the germination phase (**Figure 8C**).

## Discussion

In this study, we studied the changes in glycerolipid profiles of *Nicotiana tabacum* pollen during the two crucial developmental stages: the germination of the hydrated pollen grain and the subsequent tip growth of the elongating pollen tube (Kim et al., 2019; Scholz et al., 2020). We demonstrated a tremendous diversity in glycerolipid composition during the pollen hydration, germination, and elongation of the emerged pollen tube. While the lipid composition of dry pollen, which includes a mixture of unique galactolipids, glycerophospholipids, sphingolipids, and sterols, is well established across many plant species (Bashir et al., 2013; Ischebeck, 2016), the studies focused on the dynamical lipidome changes in non-stress conditions are still scarce (Dorne et al., 1988). Our data highlight the (apparent) importance of the pollen germination phase, which is often skipped or overlooked in functional pollen studies, especially those concerned with membrane-related processes. We argue that during pollen germination, an unparalleled combination of massive lipid metabolism activation and tremendous membrane trafficking, which also includes many lipid signaling pathways, coexists together for a relatively short time (Žárský et al., 2006; Sekereš et al., 2015). While it is evident that the *de novo* synthesis in the ER represents the main portion of structural phospholipids in every whole cell lipidomic study, our microscopic and *in situ* phospholipase activity data suggests that processes beyond lipid biosynthesis may be responsible for a significant portion of observable lipid changes.

In this regard, PA represents a great example of theoretical and methodological challenges connected with lipidomic studies: PA is a critical component of phospholipid biosynthesis and an important signaling molecule. In plant cells, PA is produced either in ER from glycerol-3-phosphate (G3P) by the sequential action of G3P-acyl transferase and lysophosphatidic acid acyl transferase (LPAAT) or at the plasma membrane (and/or Golgi or endosomal compartments) *via* the activity of PLD or DAG kinase (DGK) (Jenkins and Frohman, 2005). The biosynthetic pathway as the major PA route in pollen was suggested recently by Hernández et al. (2020) in olive, and pollen-abundant LPAAT3 was hypothesized to play a vital role in the Arabidopsis male gametophyte (Kim et al., 2005). Conversely, both PLD and DGK pathways were repeatedly implicated in PA-dependent polar growth of pollen tubes. Plasma membrane PA generated from structural phospholipids *via* PLD was implicated in tobacco pollen tube growth (Potocký et al., 2003), and tobacco PLDδ3 was proposed as the major PM-localized isoform (Pejchar et al., 2020). Multiple reports also pinpointed the role of several DGK isoforms: in Arabidopsis, ER-localized DGK2 and DGK4 are required for gametogenesis and pollen tube growth (Dias et al., 2019; Angkawijaya et al., 2020). Tobacco DGK5 is involved in the polar secretion of cell wall material to the pollen tube tip, possibly by regulating phosphoinositide-signaling pathways (Scholz et al., 2022). Our multifaceted data on PA generation and dynamics strongly suggest that in tobacco pollen, PA production comes mainly from PC and, to a lesser extent, from PE, as can be inferred from individual species profiles. We predict that this PA route represents a significant pathway and that PLD and NPC/DGK pathways generate distinct PA cellular pools. However, great caution must be taken in interpreting PA data from pollen lipidomic studies, as non-optimal lipid isolation may lead to artificially high PA levels due to PLD release and activation. Paradoxically, this is often the case when pollen tubes are separated from the medium without great care prior to hot isopropanol treatment. Seeming PA levels in whole cell samples may reach 20-60% of total glycerolipids, as was reported for soybean, tobacco, or Arabidopsis pollen (Djanaguiraman et al., 2013; McDowell et al., 2013; Krawczyk et al., 2022). Our data, reporting whole cell PA levels at 1.5-2.3% of total glycerolipids and PA levels in the PM at ~7% of total glycerolipids, agree with the data on wheat pollen and purified plasma membrane from tobacco cell culture (Cacas et al., 2016; Narayanan et al., 2018).

In a partial analogy to the PA, the surprisingly high dynamics of lysophospholipid levels (particularly LPC, but also LPE and perhaps even LPG) during the pollen germination phase can also be attributed to both metabolic and membrane signaling/remodeling pathways. LPC can be produced as a byproduct of the last step of triacylglycerol synthesis *via* phospholipid:diacylglycerol acyltransferase (PDAT) activity or generated from PC by PLA_2_ activity. PDAT1 was indeed shown to be implicated in the proper development of Arabidopsis pollen together with diacylglycerol:acytransferase (Zhang et al., 2009). On the other hand, PDAT contribution to TAG synthesis (and thus LPC production) in olive pollen is negligible (Hernández et al., 2020). On the other hand, activities of small secretory PLA_2_ isoforms β, γ, and δ have been shown to control Arabidopsis pollen development, reportedly in the ER or Golgi. Notably, LPE was highlighted as the responsible lysophospholipid (Kim et al., 2011). Our results, showing high PLA_2_ activity during the germination phase and demonstrating massive enrichment of lysophospholipids in PM fraction, also support the role of PLA_2_ activity as the primary source of lysophospholipid production. However, our data paint a slightly different picture, as they place the site of the LPC action on the PM. Brown et al. (2003) proposed that PLA_2_ hydrolyzes the phospholipids on the extraplasmatic leaflet of the membrane, generating a localized concentration of inverted cone-shaped lysophospholipids that drive the formation of positive membrane curvature. Indeed, mammalian PLA_2_ and extracellular LPC were shown to regulate membrane-protein trafficking and exocytosis in concert with phosphatidic acid (Choukroun et al., 2000; Zeniou-Meyer et al., 2007). In plants, it has been found that LPC also contributes to the activation of the plasma membrane H^+^-ATPases (Wielandt et al., 2015).

Although the lipidomic approaches and *in situ* phospholipase assays converged on the same lipid classes and biological processes, the temporal overlap was only partial (**Figures 2**, **7**). Several, not mutually exclusive, explanations for this discrepancy can be drawn. First, both approaches tackle the lipid quantification from a slightly different angle: while lipidomics gathers steady-state levels of the lipids with great accuracy and distinguishes individual lipid species, *in situ* lipase analysis (or any other lipid pulse-labeling technique) uncovers immediate dynamical changes and fluxes (Kalachova et al., 2022). Second, the lipase activity analyses visualize specific enzyme activities, while the lipidomic approaches do not discriminate. It is thus possible that the high lysophospholipid levels detected in the imbibed pollen are either a remnant of the TAG synthesis during pollen maturation or products of phospholipase A_1_, while the rapid production of Bodipy-LPC reflects only the activity of PLA_2_. Similarly, increasing PLD activity, detected by the *in situ* approaches during pollen tube growth, may be counteracted by PA phosphatases that keep the steady state level of pollen tube PA constant.

Interestingly, the composition of galactolipids revealed distinct behavior of MGDG and DGDG (**Figure 2**). Although it is not clear if these galactolipids are strictly extraplastidial or are also present in pollen proplastids, both MGDG and DGDG are a common presence in pollen lipidomes from various species, e.g., Arabidopsis pollen contains 5% of MGDG and 2% of DGDG (McDowell et al., 2013), which roughly corresponds to our findings (1.5% for MGDG and 0.5% of DGDG in total membrane lipids). Significantly, both galactolipids were also detected in the enriched-PM fraction. While the specific function of MGDG and DGDG remain obscure, DGDG was reported to localize to the plasma membrane, and the chemical inhibition of MGDG synthase affects pollen tube growth but not germination (Botté et al., 2011). Moreover, increased galactolipid levels were linked to pollen tube growth in lily (Nakamura et al., 2009). This is consistent with our data showing the temporal decrease of DGDG during the germination phase, although the functional significance remains to be established. The same holds true for PS, another lipid class showing temporal decrease during the germination phase.

The swap between the high abundance lipids PC and PE in the transition from the germination phase to the pollen tube growth phase is unclear. It is known that changes in PC/PE ratio in mammalian cells affect membrane integrity (Li et al., 2006). Alternatively, an increase of PE vs. PC in the pollen tube growth may be linked with its function in the electrostatic/hydrogen bond switch, by which the electrostatic interactions of peripheral membrane proteins with PA at the plasma membrane is regulated (Kooijman et al., 2007). Finally, PE was suggested as the primary glycerolipid regulator of membrane fluidity upon changes of cellular sterol contents (Dawaliby et al., 2016). Given that pollen sterols show remarkable diversity (Rotsch et al., 2017) and sterols were described to regulate cell polarity cues (Han et al., 2018), versatile PE levels might be required to maintain the proper balance that would allow the existence of sterol-induced membrane nanodomains and buffer the membrane fluidity required for rapid pollen tube growth.

## Conclusions

In summary, we demonstrated that the establishment and maintenance of cellular polarity, exemplified here on the model of germinating pollen and tip-growing pollen tubes, are accompanied by complex changes in membrane glycerolipids. Such dynamics is likely an interplay of rapid metabolic changes needed by rapid cell expansion and concurrent anterograde and retrograde membrane trafficking regulated by phospholipid-derived signaling pathways. Our study thus provides a blueprint for future studies on the functional significance of individual lipids in different polarity processes and calls for analogous studies aimed at the dynamics of neutral lipids and sphingolipids.

## Data availability statement

The mass spectrometry proteomics data generated in this study are available in the ProteomeXchange Consortium with the dataset identifier PXD037046 (https://www.ebi.ac.uk/pride/archive/projects/PXD037046).

## Author contributions

MP conceived and designed the experiments. NS, PP, HS, MH, and MP carried out the experiments. MP and NS wrote the manuscript with the help of PP. All authors contributed to the discussion of the results and edited and approved the submitted version of the manuscript.

## Funding

This work was supported by the Czech Science Foundation grants GA19-21758S and 21-09254S to MP, and 20-21547S to PP; NS was supported by the Postdoctoral Fellowship Program of the Czech Academy of Sciences (L200382051) and the Ministry of Education, Youth and Sports of the Czech Republic (MEYS CR)/Charles University (OP RDE, call no. 02_18_053). The Imaging Facility of the Institute of Experimental Botany of the Czech Academy of Sciences (IEB CAS) is supported by the MEYS CR LM2018129 Czech-Bioimaging and the IEB CAS.

## Acknowledgments

The lipid analyses described in this work were performed at the Kansas Lipidomics Research Center Analytical Laboratory. Instrument acquisition and lipidomics method development were supported by the National Science Foundation (including support from the Major Research Instrumentation program; most recent award DBI-1726527), K-IDeA Networks of Biomedical Research Excellence (INBRE) of National Institute of Health (P20GM1013013), and Kansas State University.

## Conflict of interest

The authors declare that the research was conducted in the absence of any commercial or financial relationships that could be construed as a potential conflict of interest.

## Publisher’s note

All claims expressed in this article are solely those of the authors and do not necessarily represent those of their affiliated organizations, or those of the publisher, the editors and the reviewers. Any product that may be evaluated in this article, or claim that may be made by its manufacturer, is not guaranteed or endorsed by the publisher.
