# Supplementary material for: Comprehensive analysis of glycerolipid dynamics during tobacco pollen germination and pollen tube growth
Source: Front Plant Sci. 2022 Nov 8;13:1028311. doi: 10.3389/fpls.2022.1028311 (PMC9679300; doi:10.3389/fpls.2022.1028311)
Supplement: Supplementary file 1 [file DataSheet_1.docx]

Supplementary Material

# Supplementary Data

The following supplemental materials are available:

# Supplementary Figures and Tables

## Supplementary Figures


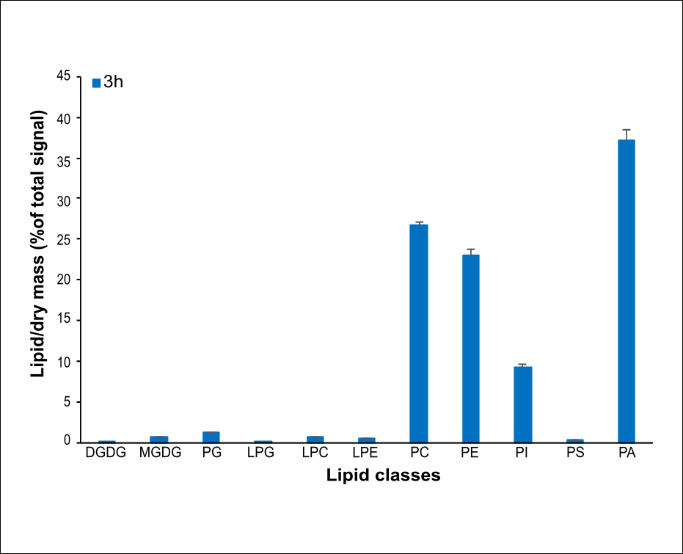


**Supplementary Figure 1.** Artificially high PA levels are measured in pollen with the hot isopropanol isolation method.


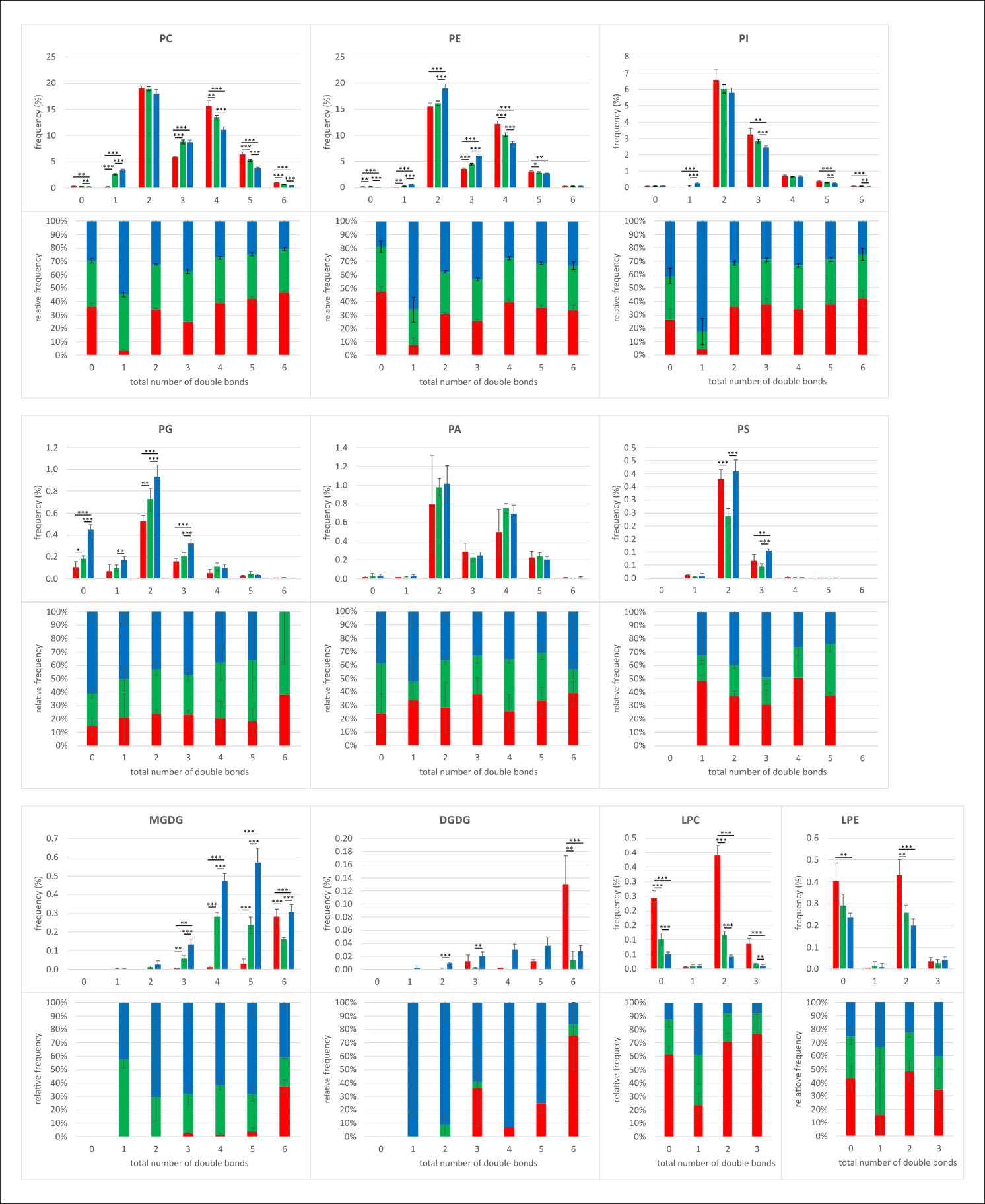


**Supplementary Figure 2.** The distribution of double bounds in individual lipid classes during pollen germination and pollen tube growth.

**
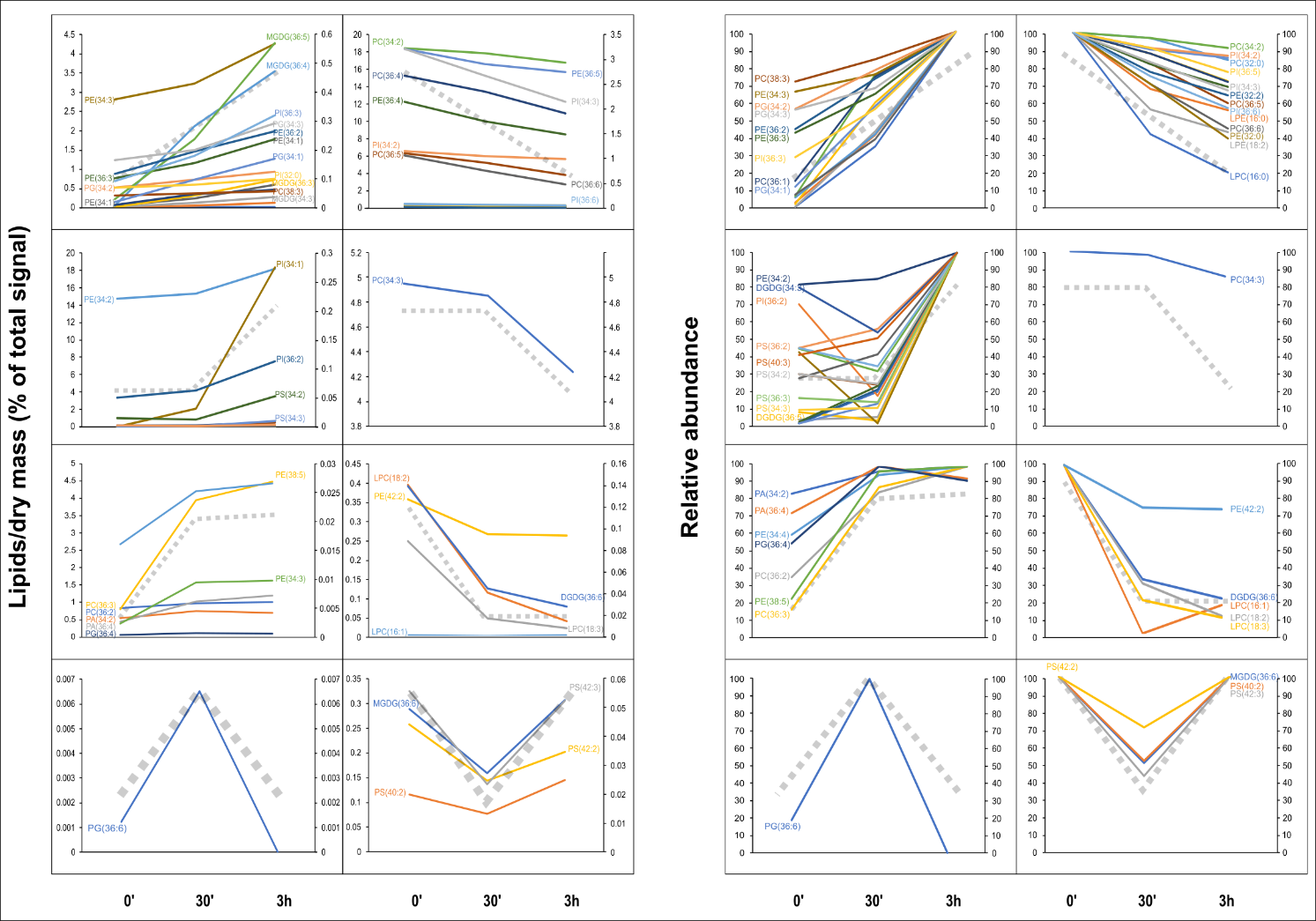
**

**Supplementary Figure 3.** Correlation pattern analysis of the individual glycerolipid species. Each pattern represents a typical trend of lipid species abundance during pollen germination and pollen tube elongation. The individual patterns are indicated as light gray dashed lines. The lipid species

following individual patterns were identified using MetaboAnalyst PatternHunter method using a predefined profile and using Pearson R to measure the distance.


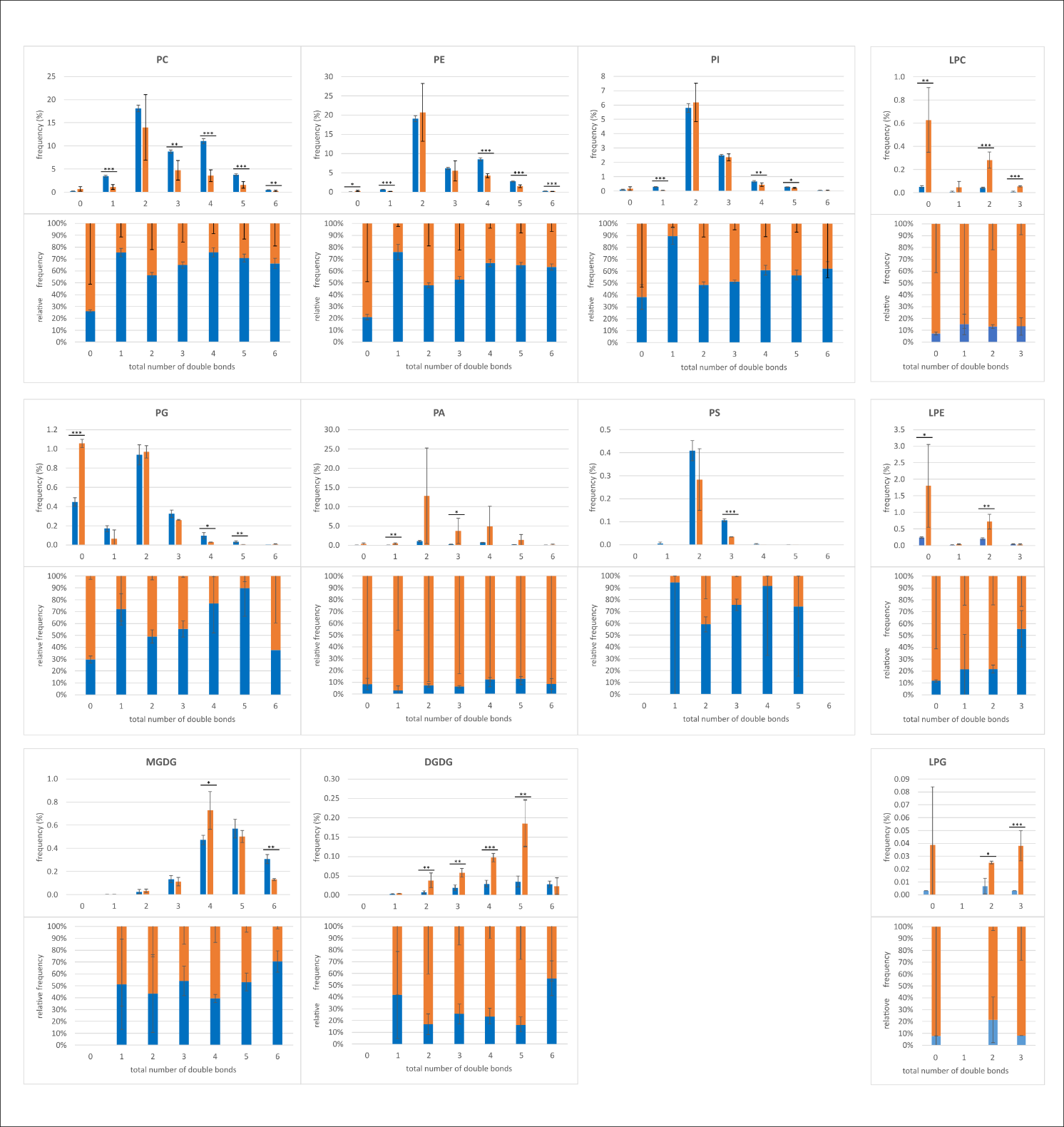


**Supplementary Figure 4**. A comparative analysis of doble bonds in individual lipid classes between plasma membrane-enriched fraction and whole cells.
